# Techniques for assessing knee joint pain in arthritis

**DOI:** 10.1186/1744-8069-3-8

**Published:** 2007-03-28

**Authors:** Volker Neugebauer, Jeong S Han, Hita Adwanikar, Yu Fu, Guangchen Ji

**Affiliations:** 1 Department of Neuroscience & Cell Biology, The University of Texas Medical Branch, 301 University Blvd. Galveston, TX 77555-1069, USA

## Abstract

The assessment of pain is of critical importance for mechanistic studies as well as for the validation of drug targets. This review will focus on knee joint pain associated with arthritis. Different animal models have been developed for the study of knee joint arthritis. Behavioral tests in animal models of knee joint arthritis typically measure knee joint pain rather indirectly. In recent years, however, progress has been made in the development of tests that actually evaluate the sensitivity of the knee joint in arthritis models. They include measurements of the knee extension angle struggle threshold, hind limb withdrawal reflex threshold of knee compression force, and vocalizations in response to stimulation of the knee. A discussion of pain assessment in humans with arthritis pain conditions concludes this review.

## Review

Arthritis represents one of the most prevalent chronic health problems and is a leading cause of disability. More than 40 million people in the United States have arthritis or chronic joint symptoms that are often accompanied by joint pain [1]. By the year 2020, this number is expected to reach 60 million. The most common form of arthritis is osteoarthritis affecting an estimated 21 million adults in the United States. Other common arthritic conditions include rheumatoid arthritis (about 2.1 million people in the United States) and gout [2]. The assessment of arthritic pain is of critical importance for the better understanding of underlying mechanisms and for the evaluation of therapeutic targets. Different animal models of arthritis are available for the assessment of joint pain and analgesic drug effects. This review will focus on arthritis models of knee joint pain and on behavioral tests used in these models. Information about the assessment of knee joint pain in humans with arthritis will also be provided.

Discussing the merits of electrophysiological studies of nociceptive processing in arthritis pain models is beyond the scope of this article. Arthritis pain-related electrophysiological changes have been measured in primary afferent nerve fibers [peripheral sensitization; 3] and in central nervous system neurons (central sensitization), including neurons in the spinal dorsal horn [4], spinal trigeminal nucleus [5], pain modulating brainstem centers [6], ventrobasal thalamus [7], somatosensory cortex [8] and amygdala [9]. While electrophysiological studies are important and necessary for the analysis of pathways, circuitry, neuronal plasticity, transmitter action and signal transduction mechanisms, behavioral tests are needed for the assessment of pain.

## Arthritis pain models

Arthritis is the inflammation of a joint, which can include infiltration of inflammatory cells (monocytes), synovial hyperplasia, bone erosion and new bone formation, narrowing of the joint space, and ankylosis of the joint [10]. The most common form of arthritis is osteoarthritis. Osteoarthritis is a degenerative disease characterized by damage to the articular cartilage, changes in subchondral and marginal bone, synovitis and capsular thickening, typically affecting weight bearing joints (knee and hips) [11]. Pain in osteoarthritis is localized and use-related, occurring during movement or weight bearing [12-14]. Rheumatoid arthritis is an autoimmune disease of the synovium that leads to an inflammatory poly-arthritis. It is characterized by the symmetrical pattern of affected joints and by morning stiffness, joint swelling and tenderness. Pain in rheumatoid arthritis improves with movement [15,16]. Gout represents one of the most painful forms of arthritis. A metabolic disorder with high blood levels of uric acid (hyperuricemia), gout is characterized by recurrent episodes of acute arthritis resulting from deposits of needle-like crystals of uric acid in the joints. The metatarsophalangeal joint (big toe) is typically affected, but other joints can be involved as well, including the knee [17,18]. The following animal models have been developed to investigate the pathophysiology of different forms of knee joint arthritis. They are also used for the assessment of joint pain but not all of them have a proven track record of predictability for human disease.

### Osteoarthritis

Animal models include spontaneous osteoarthritis in specific strains (mouse and guinea pig) and osteoarthritis induced chemically or mechanically (surgically) [12-14]. Chemical models involve intra-articular injections of compounds that cause joint pathology through inhibition of chondrocyte metabolism by papain or monosodium iodoacetate (MIA) and damage of ligaments and tendons with collagenase. Surgical models induce joint instability by (partial) meniscectomy combined with transection of collateral and/or cruciate ligaments [12-14]. The MIA model has emerged as a particularly useful osteoarthritis model for the study of pain and analgesic drug effects because it is reproducible and mimics pathological changes and pain of osteoarthritis in humans. Intraarticular injection of MIA produces progressive joint degeneration through inhibition of glycolysis and subsequent chondrocyte death that develops over several weeks. Similar to human osteoarthritis, joint pathology is characterized by chondrocyte necrosis resulting in decreased thickness of the articular cartilage and fibrillation of the cartilage surface, separation of the necrotic cartilage from the underlying bone and exposure of the subchondral bone; osteolysis and swelling; and reductions in bone mineral content and density [13,14,19].

### Inflammatory mono-arthritis

Recurrent inflammatory phases are common in human osteoarthritis [1]. The acute inflammatory phase of osteoarthritis is also mimicked by the kaolin/carrageenan-induced knee joint arthritis model (K/C arthritis). Intraarticular kaolin and carrageenan injections into one knee produce an aseptic use-dependent monoarthritis with damage to the cartilage, inflammation of the synovia and synovial fluid exudate. The K/C arthritis develops rapidly within hours and persists for weeks. Pathological, behavioral and electrophysiological changes have been studied extensively in the K/C arthritis model in mouse, rat, cat and non-human primate [20-25]. A modification of the K/C arthritis model is the knee joint monoarthritis induced by intraarticular injection of carrageenan alone. The time course of the carrageenan-arthritis is shorter (hours to days) and the cartilage damage less pronounced than in the K/C model [26-29].

Other models of inflammatory mono-arthritis in the knee include the acute zymosan-induced arthritis and the chronic complete Freund's adjuvant (CFA) induced arthritis. Injection of zymosan into one knee produces an erosive synovitis in mouse and rat. The zymosan arthritis is characterized by an acute phase of increased vascular permeability, edema formation, neurophil infiltration and exudate within hours, whereas the chronic phase (days to several weeks) resembles chronic rheumatoid synovitis with mononuclear cell infiltration (macrophages and lymphocytes), fibroblast reaction and pannus formation [30-32]. The CFA mono-arthritis of the knee is induced by intraarticular injection of complete Freund's adjuvant, suspension of heat-killed *Mycobacterium butyricum or Mycobacterium tuberculosum *[33-36]. This chronic monoarthritis is characterized by joint inflammation, cartilage destruction and bone erosion, which persist for at least several weeks. The CFA mono-arthritis model represents a modification of the classical adjuvant-induced poly-arthritis. While the CFA model is well established in rats, it has been difficult to produce a reliable CFA arthritis in mice. Only recently a CFA-arthritis model was developed in mice in which repeated injections of a much higher concentration of CFA into one knee (once per week for 4 weeks) produced a monoarthritis of the knee with synovial hypertrophy, neutrophil infiltration, mild erosion of cartilage and bone, and small amounts of pannus [37]. The murine CFA-monoarthritis lasted for at least 5 weeks after the first intraarticular injection whereas a single injection produced a short-lasting inflammation that resolved within 7 days.

### Rheumatoid Arthritis

Animal models include poly-arthritis induced by immunogenic adjuvants (CFA and cartilage antigens) and non-immunogenic adjuvants (lacking bacterial cell wall or peptide-containing components) [15,38]. The knee joint is not the primary target and area of interest in these models. Further, the systemic nature of this arthritis may affect the overall condition and well being of the animals and may confound pain assessment. The widely used CFA poly-arthritis represents a model of chronic immune-mediated joint inflammation that is induced by intradermal or subcutaneous injection of a suspension of heat killed *Mycobacterium butyricum or Mycobacterium tuberculosum *(CFA) at the base of the tail or in the foodpad. The ensuing poly-arthritis represents a systemic disease with inflammation of distal joints of the limbs (ankle, wrist, tarsal, carpal, interphalangeal joints) and spinal joints, lesions of the eyes, ears, nose, skin and genitourinary and gastrointestinal tracts, as well as anorexia and profound weight loss [3,15]. The CFA arthritis follows a biphasic time course, consisting of an acute local inflammatory reaction within hours that subsides after 3–5 days and a chronic systemic reaction that shows a relapsing-remitting course after the initial two weeks and can persist for several months. The persistent disease ultimately results in chronic joint deformation and signs of joint destruction, including synovitis and synovial hyperplasia, angiogenesis, pannus formation, capsular fibrosis, cartilage destruction, bone erosion and new periosteal bone formation, bone matrix resorption, inflammation of the bone marrow, and ankylosis [39]. The CFA poly-arthritis model is well established and reproducible in rats but not in mice (however, see CFA mono-arthritis).

Another immunogenic adjuvant model of rheumatoid arthritis model is induced by cartilage-derived proteins such as collagen II, collagen XI and cartilage oligomeric matrix protein (COMP) in rat and mouse [38]. Emulsified with CFA the cartilage antigens are injected intradermally at the base of the tail. The best characterized cartilage-induced arthritis model is the collagen type II arthritis (CIA arthritis), which leads to a severe erosive poly-arthritis affecting the hind paws and knees [40,41]. Periarticular erythema and edema and neutrophil infiltration appear in the hind paws after 2–3 weeks followed by a chronic relapsing phase (5 weeks) when the severity of arthritis progresses to include pannus formation, erosion of cartilage, bone resorption, osteophyte formation, restructuring and ankylosis of the joints [38,40,42]. The autologous collagen type XI-induced arthritis shows a more aggressive course and pathology, whereas the COMP-induced arthritis is rather acute and self-limited [38].

Adjuvant arthritis without an autoimmune component is induced by compounds which do not contain major histocompatibility complex binding peptides but involve T-cell activation. These "pure" adjuvants include mineral oil (incomplete Freund's adjuvant), avridine, squalene and pristane [38]. Intradermal or subcutaneous injections of pure adjuvants produce a chronic relapsing arthritis with characteristics of rheumatoid arthritis. The severe and long-lasting (months) arthritis appears after 1–2 weeks in peripheral joints, mainly in the hind limbs, with pannus formation, erosion of cartilage and bone, and joint deformation [38].

### Gouty arthritis

Gout is a metabolic disease characterized by recurrent episodes of acute arthritis in the metatarsophalangeal joint but can also affect the knee joint [17,18]. Gouty arthritis results from the deposits of needle-like crystals of uric acid in the joints, causing inflammation with severe pain in the affected joint. Injection of monosodium urate crystals dissolved in saline [43] or uric acid suspended in mineral oil [17] into the knee joint leads to an acute inflammation (synovitis) within 2–3 hours, which persists at maximum levels for hours and resolves after 3–7 days

## Pain behavior of arthritic animals

The main challenge of assessing knee joint pain has been to develop tests that actually measure the sensitivity of the knee joint rather than that of the hind paw [33]. Behavioral tests that use indirect measures of knee joint pain in arthritis models include static and dynamic weight bearing [13,26,43-46]; foot posture [43,47] and gait analysis [43,48], including paw elevation time during walking [17,27,32,49]; spontaneous mobility [50,51]; and mechanical or heat sensitivity of the paw [14,45-47,52]. Though indirect measures, weight bearing and gait analysis have the advantage that they are also used in the clinical setting to assess pain in patients with arthritis (see "Pain assessment in patients with arthritis").

More recently, behavioral tests have been developed that directly assess the mechanical sensitivity of the knee by measuring the hind limb withdrawal reflex threshold of knee compression force [33,37,51,53], struggle threshold angle of knee extension [33,35], and vocalizations evoked by stimulation of the knee [33,51,54].

### Weight bearing

**Figure 1:**
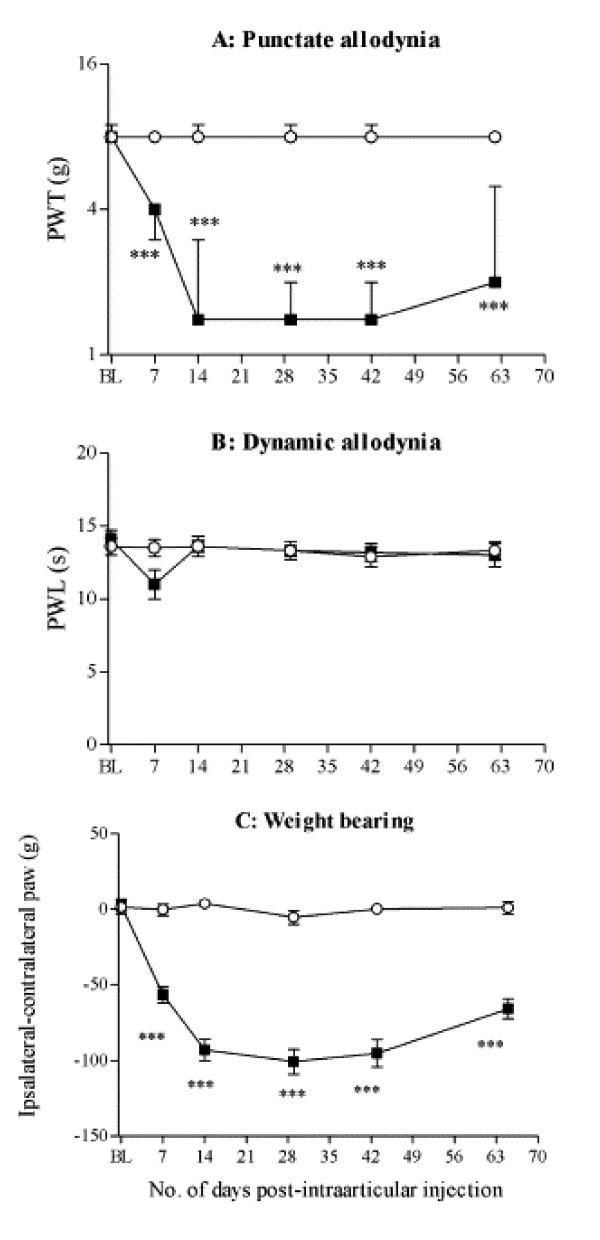
Development of punctate (A) and dynamic (B) allodynia and weight bearing deficit (C) following intraarticular injection of monosodium iodoacetate (MIA, 2 mg; ■) MIA or saline (○) in the right knee. (A) Baseline (BL) paw withdrawal thresholds (PWT) were determined in both hind paws prior to injection. PWT to von Frey hair stimulation of the plantar paw surface were assessed on various days post-injection. Results are expressed as median force (g) required for a paw withdrawal in 10 animals per group (vertical bars represent first and third quartiles). * *P *< 0.05, ** *P *< 0.01, *** *P *< 0.001 significantly different (Mann-Whitney *U *test) from saline-treated group at each time point. (B) Baseline (BL) paw withdrawal latencies (PWL) to stroking the plantar paw surface with a cotton bud were determined for both hind paws prior to injection. Results are expressed as mean PWL (s) in 10 animals per group (vertical bars represent ± SEM). * *P *< 0.05, ** *P *< 0.01, *** *P *< 0.001 significantly different (one-way ANOVA followed by Dunnett's posthoc test) from saline-treated group. (C) Baseline (BL) hind paw weight distribution was determined prior to injection. Changes in hind paw weight distribution were assessed on various days post-injection. Results are expressed as mean change in weight distribution (contralateral-ipsilateral) (g) in 10 animals per group (vertical bars represent ± SEM). * *P *< 0.05, ** *P *< 0.01, *** *P *< 0.001 significantly different (one-way ANOVA followed by Dunnett's posthoc test) from saline-treated group. Reprinted from [46], Copyright 2004, with permission from Elsevier.

Measurements of weight bearing have been used in mono-arthritis models induced in the knee joint by carrageenan, urate, MIA or papain and by surgery (partial meniscectomy). Most commonly, the weight distribution on the two hind paws is measured as the force exerted by each limb on a transducer plate in the floor over a given time period [13,26,43-46]. Weight borne by each hind limb is expressed as percent of body weight [14,44] or percent of weight borne by both hind limbs [13,45]. The ratio [19,43] or difference [46,55] of weight distribution (force) between each hind limb are also calculated. A significant shift of weight from the arthritic site to the contralateral limb, i.e., a weight-bearing deficit, is taken as a pain measure and has been shown in knee joint arthritis models induced by intraarticular MIA [13,14,45,46,55], papain [13], urate [43] and carrageenan [44] and by partial meniscectomy [14,56]. Figure 1 illustrates the weight bearing deficit in rats with MIA-induced knee joint arthritis. These static measurements of weight bearing by the hind limbs typically involve restraining the animals and do not assess the shift of weight distribution to the forelimbs as occurs with hind limb injury such as arthritis [48].

Weight bearing across all four limbs has been measured in rats with a carrageenan-induced knee joint arthritis [26,29]. Weight load on each limb is detected while the animal is walking across four pairs of force sensor plates in the floor of an enclosed walkway. Time-weight curves for left and right fore limbs and hind limbs of rats with arthritis show a reduction of weight load on the affected limb for up to one week. Similarly, weight distribution across the four limbs has been measured in the MIA arthritis model using load cell platforms in two sections of the glass floor in the central portion of a chamber [48]. The digitized load cell output and simultaneously videotaped images are used to calculate the peak vertical load bearing by each limb. Load bearing by the affected limb is reduced for several weeks. Weight distribution across the four limbs has also been determined with a gait analysis system ("CatWalk") that measures the intensity of the illumination caused by paw contact with a glass floor [57]. The intensity correlates with pressure (weight support) and mechanical withdrawal thresholds and is significantly reduced in the affected limb of neuropathic rats. The CatWalk analysis system may also be useful for the assessment of weight-bearing in arthritis. A potential problem with dynamic weight bearing measurements is that animals are required to move, which can be influenced by a number of factors such as motivation (see mobility).

### Posture and gait analysis

Related to the assessment of weight bearing, abnormal posture of the hind paw and gait have been quantified in knee joint arthritis models using subjective rating scales. Static (standing) and dynamic (walking) behaviors have been analyzed separately to calculate a "pain score" in rats with urate-induced knee joint arthritis [43]. Categories of the rating scale include complete touch of foot pad, partial touch or one foot stand (standing position) and slight limping, severe limping or one foot gait (walking state). A combination of posture and gait analysis has been used to rate pain-related spontaneous behavior in the carrageenan-induced knee joint arthritis [47]. Behavioral signs include curling toes, eversion of the foot, partial weight bearing, non-weight bearing and guarding, and avoiding contact with the limb.

Gait disturbance has also been detected using the knee joint incapacitation test in rats with knee joint arthritis induced by intraarticular injections of carrageenan [27], zymosan [32,49] or uric acid [17]. Increased paw elevation times are measured in arthritic rats walking on a rotating mesh-covered steel drum. Metal gaiters ("electrodes") wrapped around the hind paws are connected via a simple circuit to a computer to record the time of contact between each hind paw and the cylinder, which is when the circuit is closed. Paw elevation time or the ratio of time of contact of the affected foot and the control foot serve as indicators of pain-related functional impairment. The advantage of this gait analysis test is that the quantitation (paw elevation time) is independent of the observer. Interestingly, temporal aspects of gait were not impaired in rats with a MIA knee joint arthritis when velocity of locomotion, stride, stance and swing times, and stride length were measured from the pattern of paw contact with the illuminated glass floor of a behavioral chamber [48].

### Spontaneous mobility

Locomotor activity has been measured in arthritic rats using biotelemetry or activity boxes. The biotelemetry system comprises a transmitter implanted in the peritoneal cavity of the rodent, and a receiver beneath the cage. The receiver detects the radio waves and activity of the rodents as counts which are registered in the computer system [50]. Loss of spontaneous mobility has been detected in rats with knee joint arthritis induced by MIA, papain, collagenase or surgical ligament transection. All models show a transitory "primary" loss of mobility for about two days after arthritis induction, presumably related to pain; but only the MIA arthritis results in a prolonged secondary loss of mobility for more than four weeks due to pain and loss of function [50]. Spontaneous exploratory activity has been measured using activity boxes that are divided in zones by photobeams consisting of pairs of infra red Light Emitting Diodes (LEDs) and phototransistors. Frequency and pattern of photobeam interruption by the animal's movements are recorded on a computer. Rats with K/C arthritis show decreased entries (number of movements from one zone to another) and increased resting time (total time during which no movements occurred) [51].

### Mechanical or heat sensitivity of the paw

Von Frey filaments and a modified Randall-Selitto analgesiometer have been used to assess the mechanical sensitivity of the hindpaw in animals with knee joint arthritis. Typically, paw withdrawal thresholds (PWT) are measured in response to increasing pressure stimuli applied to the plantar surface by von Frey filaments or to the dorsal surface by a wedge-shaped probe of a Randall-Selitto analgesiometer. Rats with knee joint arthritis induced by MIA [14,45,46] have decreased PWT (mechanical allodynia; see Figure 1) for several weeks on the affected limb measured with either technique, but show little dynamic allodynia assessed by stroking the plantar surface of the paw. Surgically induced knee joint arthritis appears to be more sensitive to von Frey hair testing than to Randall-Selitto analgesiometry [14,56]. Bilateral decreases of mechanical PWT measured with von Frey filaments occur in the K/C knee joint arthritis model [52].

Thermal sensitivity of the paw has been measured in arthritic rats using the hot-plate test and the paw withdrawal latency (PWL) to noxious heat. A unilateral decrease of PWL is observed in rats with a K/C knee joint arthritis [20,24,47]. Heat hyperalgesia was observed for less than 48 hours by one group [24,47] but lasted for at least two weeks in another study [20], which would be consistent with the prolonged time course of the K/C arthritis as mentioned earlier [see 33]. Mice with a K/C knee joint arthritis also show decreased PWL but unchanged hot-plate latency, which is the time until the animal shakes or licks its hind limb [25]. No thermal hyperalgesia has been found in the surgically induced knee joint osteoarthritis model [56].

These tests assess secondary hyperalgesia or allodynia, which has been reported in patients with osteoarthritis but is not very common [13]. The following direct measures of knee joint pain have been developed recently.

### Mechanical sensitivity of the knee

The threshold for hind limb withdrawal reflexes evoked by compression of the knee has been measured in arthritic rats and mice [37,51,53]. The knee joint of rats is compressed with a calibrated forceps equipped with force transducers (strain gauges) whose output is amplified, digitized and recorded on a computer and/or displayed in grams on a liquid crystal display screen [33,51,53]. Hind limb withdrawal reflexes in mice have been assessed by scoring the intensity of the manual compression of the knee required to evoke the reflex [37]. Withdrawal thresholds for stimulation of the arthritic, but not the contralateral, knee decrease in rats with K/C arthritis [51,53] and in mice with CFA-induced knee joint arthritis [37]. This may be an important difference to the measurement of secondary mechanical allodynia in the PWT test that has shown bilateral changes in the K/C knee joint arthritis model [52].

### Struggle threshold angle of knee extension

**Figure 2:**
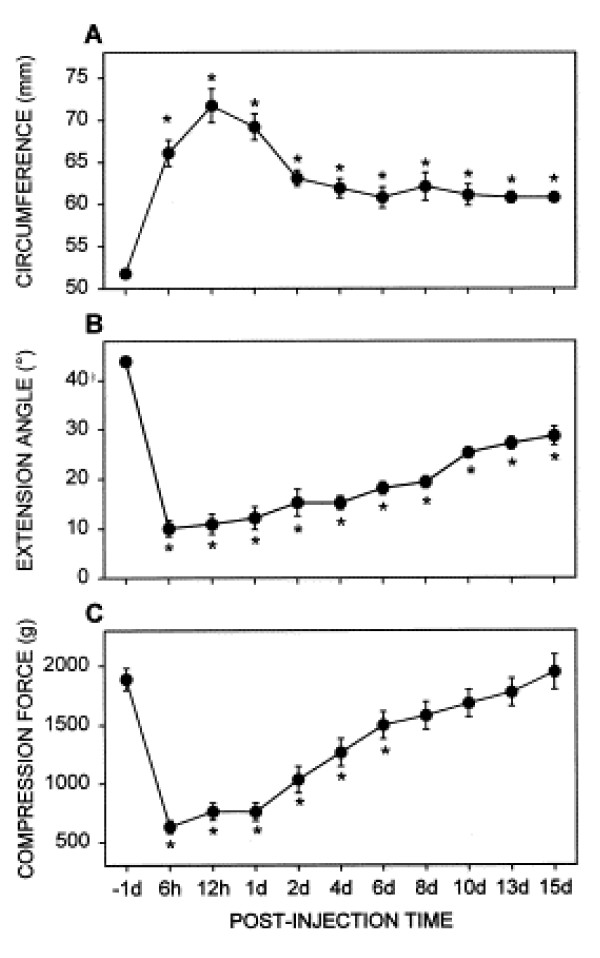
Time course of the changes of three outcome measures in rats with a kaolin/carrageenan (K/C)-induced arthritis. (A) Circumference of the knee before and after K/C injection. (B) Angle at which the knee could be extended before eliciting struggling behavior in the rat. (C) Vocalization threshold of the compression force, which was applied to the knee. Post-injection time is expressed as hours (h) or days (d) after K/C injection. Pre-injection control was taken one day before the injection (-1 d). Asterisks indicate values significantly different from the pre-injection control value by one-way ANOVA followed by the Dunnett's posthoc test (n = 10). Symbols and error bars represent mean ± SE. Reprinted from [33], Copyright 2002, with permission from Elsevier.

Reduced range of motion and mechanical sensitivity of the arthritic knee have been assessed by measuring the struggle threshold of the knee extension angle [33,35]. In this quantitative test the tibia is extended until the rat shows struggling behavior, while the femur is held in position. The extension distance that the heel travels during movement is measured to calculate the extension angle by a trigonometric function that uses the length of the tibia and extension distance. In rats with knee joint arthritis induced by K/C [33] or CFA [33,35] the struggle threshold angle of the extension of the arthritic knee is decreased compared to the contralateral knee for nearly two weeks in the K/C model and for 2–3 weeks in the CFA model. Figure 2 shows the time course of decreased struggle threshold in the K/C arthritis model.

### Vocalizations evoked by compression of the knee

**Figure 3:**
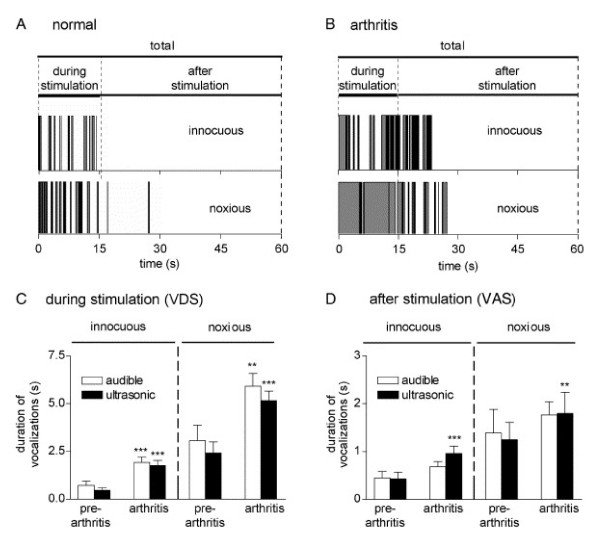
Increased audible and ultrasonic vocalizations in the K/C model of arthritic pain. (A, B) Original recordings of ultrasonic vocalizations evoked by innocuous (upper trace) and noxious (lower trace) stimulation of the knee joint in a rat before (A) and after (B) induction of arthritis with intraarticular kaolin and carrageenan injections. Mechanical stimuli were applied for 15 s; duration of the recording period was 1 min. Vocalizations during and after stimulation (VDS and VAD, respectively) were analyzed separately. (C) Duration of audible and ultrasonic VDS increased significantly 6 h after induction of arthritis compared to the values measured in the same animals before arthritis (n = 16). Stimuli of innocuous (left side) and noxious (right side) intensities evoked VDS of longer duration in arthritic animals compared to controls. (D) Duration of ultrasonic, but not audible, VAD following innocuous (left) and noxious (right) stimuli increased significantly in the arthritis pain model (6 h postinduction; n = 16). Symbols and error bars represent mean ± SE. ** P < 0.01, *** P < 0.001. Reprinted from Han JS & Neugebauer V [54]. PAIN 2005;113-211-222. Used with permission from the International Association for the Study of Pain^®^.

Rodents vocalize in the audible and ultrasonic ranges. When evoked by noxious stimuli, audible vocalizations represent a nocifensive reaction whereas ultrasonic vocalizations in the 22 kHz range reflect an emotional-affective response [51,54]. The threshold of audible vocalizations has been measured by compressing the knee of manually restrained rats with a calibrated forceps as described above [33]. Vocalization thresholds are significantly decreased for one week in rats with a K/C arthritis (see Figure 2) and for two weeks in the CFA knee joint arthritis model [33]. A recording chamber and computerized analysis system has been developed to measure simultaneously audible and ultrasonic vocalizations evoked by stimulation of the knee [51,54]. Rate and duration of audible and ultrasonic vocalizations are increased in rats with a K/C knee joint arthritis [51]. It should be noted that the functional relationship between audible and ultrasonic vocalizations, which are generated by different neural mechanisms, has not been addressed in these studies. Vocalizations that occur during stimulation (VDS) and vocalizations that outlast the stimulus (vocalization afterdischarges, VAD) have also been analyzed separately. VDS are organized in the brainstem at the medullary level whereas VAD are organized in the limbic forebrain, including the amygdala. Figure 3 shows that both VDS and VAD increase in rats with a K/C arthritis [54].

## Pain assessment in patients with arthritis

Mechanical pain thresholds, range of motion, weight bearing and gait analysis have been measured in patients with knee joint arthritis. In addition, a variety of patient self-report questionnaires are frequently used to assess pain and function in patients with arthritis, including the Visual Analog Scale (VAS) and other rating scales, McGill Pain Questionnaire (MPQ) and its short form (SF-MPQ), Western Ontario and McMaster Universities Osteoarthritis Index (WOMAC), Health Assessment Questionnaire (HAQ), Medical Outcomes Study 36-Item Short-Form Health Survey (SF-36), and Disease Activity Score (DAS 28) [58].

### Pain thresholds

**Figure 4:**
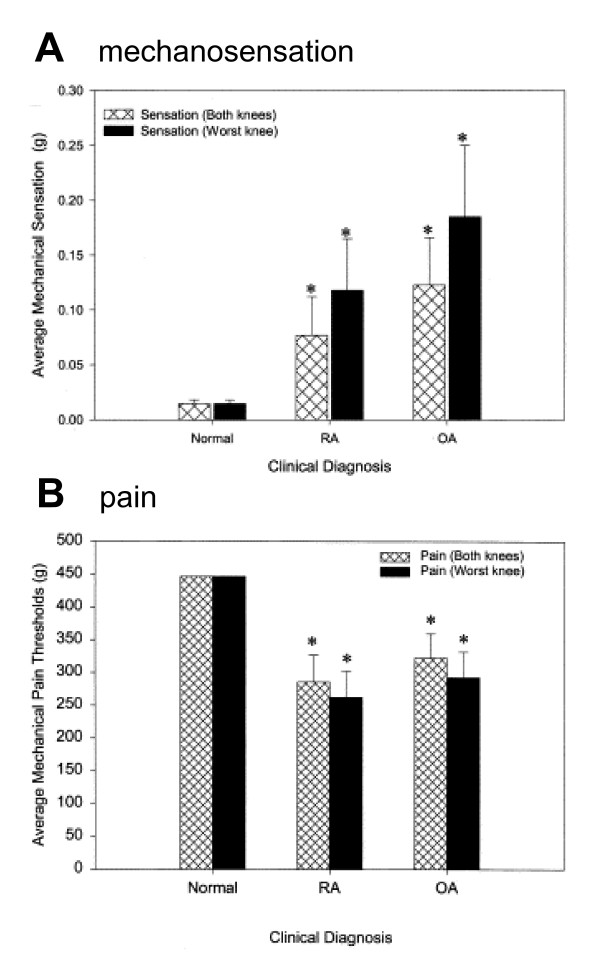
Altered thresholds for mechanosensation (A) and pain (B) in patients with rheumatoid arthritis and osteoarthritis. (A) Mechanical sensation thresholds (g) for normal (Norm), rheumatoid arthritis (RA) and osteoarthritis (OA) patients, determined by von Frey monofilament testing. The RA and OA groups had significantly higher average mechanical sensation thresholds in both knees. Monofilament diameter scores for each knee were converted to grams per protocol convention for threshold determination. Average and standard error mechanical sensation scores for both knees and for the most symptomatic (worst) knee are demonstrated. * P *<*0.05 when compared to normal controls, analyzed by paired and unpaired Student t-tests. (B) Mechanical pain threshold scores (g) for Norm, RA and OA patients, determined by von Frey monofilament testing (see A). The average pain threshold for both knees and for the most painful (worst) knee was significantly lower in RA patients than in the Norm group. The average pain threshold for both knees and for the most painful knee in OA patients was significantly lower than in the Norm group. * P < 0.05 when compared to normal controls, analyzed by paired and unpaired Student t-tests. Reprinted from [59], Copyright 2003, with permission from Elsevier.

Mechanical thresholds for pain are decreased in patients with osteoarthritis or rheumatoid arthritis [59]. Figure 4 shows the results obtained with von Frey filaments applied to the arthritic knee similar to studies in animals (see above). Whereas patients with osteoarthritis or rheumatoid arthritis have increased thresholds for mechanosensation, their cutaneous pain thresholds are lower than those of normal subjects [59].

### Range of motion and stiffness

An ultrasonic device has been used to measure the position of external markers attached to anatomical reference points of the legs in the pendulum test of Wartenberg [60]. Patients with rheumatoid arthritis show increased knee stiffness (damping ratio) and reduced maximal amplitude of flexion and extension. Reduced range of motion has also been measured in patients with knee osteoarthritis [61].

### Weight bearing and gait analysis

In patients with knee osteoarthritis, the percentage of pressure on forefoot and hindfoot (static pedobarography) and the peak pressures on forefoot, midfoot, and hindfoot (dynamic pedobarography) have been measured using a force platform with independent pressure-measuring cells [62]. Analysis of the pressure map showed that the percentage of hindfoot pressure during standing and peak pressure of the forefoot during walking are lower in patients with osteoarthritis, reflecting weight bearing changes. Spatiotemporal and kinematic data of patients with knee osteoarthritis have been obtained using a computerized gait analysis system with video cameras [63]. External reflective markers are placed on anatomical reference points of the legs to determine the limb position, and two force plates measure ground reaction forces. In the osteoarthritis group, walking velocity, cadence (steps/min) and stride length are reduced, stride time and double support time are increased, and the overall stance phase is prolonged. Knee flexion during stance and swing phases is reduced. Extensor and flexor moments (Nm/kg) are also altered and the peak values of ground reaction forces are lower, suggesting gait changes in osteoarthritis patients.

### Pain assessment in self-report questionnaires

The Visual Analog Scale (VAS) has been used in patients with osteoarthritis and rheumatoid arthritis affecting the knee [59]. Other pain scales include numeric rating scales and the Neuropathic Pain Scale (NPS) for osteoarthritis [64]. The McGill Pain Questionaire (MPQ) uses word descriptors (sensory, affective and evaluative) and an intensity scale to generate three pain scores, the pain rating index, number of words chosen and Present Pain Intensity (PPI) index [65]. The Short-Form McGill Pain Questionnaire (SF-MPQ) includes sensory and affective descriptors, which are rated on an intensity scale to generate three pain scores (sensory, affective and total). The SF-MPQ also includes the PPI index of the standard MPQ and a VAS [66]. MPQ and SF-MPQ have been used for pain assessment in patients with arthritis.

The Western Ontario and McMaster Universities Osteoarthritis Index (WOMAC) is one of the most commonly used measures of pain and physical disability in patients with osteoarthritis of the hip and/or knee [67,68]. Its reliability and validity have been demonstrated in a range of patient groups and interventions. The WOMAC evaluates three dimensions (pain, stiffness and physical function) using a numeric rating scale (Likert version) or VAS. In addition to the score of each subscale, an index score or global score is calculated.

The Brief Pain Inventory (BPI) is an established tool for the assessment of cancer pain and has recently also been used in patients with osteoarthritis [69]. The BPI includes two numerical rating scales that assess the severity of pain and the impact of pain on daily functions (interference). The four-item severity subscale asks patients to rate their worst pain, least pain, average pain over the previous 24 hours, and pain right now. The seven-item interference subscale of the BPI assesses general activity, walking ability, normal work, mood, sleep, relations with people, and enjoyment of life. A modified BPI short form assesses three pain severity items (worst pain, pain on the average, and pain right now) and five interference items (walking ability, mood, sleep, relations with others, and ability to concentrate) [70].

The Health Assessment Questionnaire (HAQ) and its derivatives have been used in patients of osteoarthritis and rheumatoid arthritis as a predictor of functional and work disability, costs, joint replacement surgery, and mortality [71,72]. The full HAQ assesses five dimensions (disability, pain, medication effects, costs of care, and mortality) with a scale of 20 activities of daily living (ADL) in eight categories and a VAS for pain. The short HAQ contains only the HAQ Disability Index (HAQ-DI) and the patient global and pain visual analog scales (VAS). Several modifications of the HAQ have been developed [58]. The multidimensional HAQ (MDHAQ) includes additional ADL and three psychological items concerning sleep, anxiety and depression. The clinical HAQ (CLINHAQ) includes anxiety and depression scales, a pain diagram, fatigue scale and other scales.

The Medical Outcomes Study 36-Item Short-Form Health Survey (SF-36) is a generic, non-disease-specific questionnaire, which includes eight scales that assess limitations in physical activities, limitations in social activities, limitations in usual role activities because of physical problems, pain, general mental health (psychological distress and well-being), limitations in usual role activities because of emotional problems, vitality (energy and fatigue), and general health perceptions [73]. The Arthritis-Specific Health Index (ASHI) for the SF-36 includes the eight-scale SF-36 and five arthritis-specific measures of knee pain on weight bearing, time to walk 50 feet, physician global evaluation of symptom severity and impact, patient global evaluation of symptom severity and impact, and pain intensity VAS [74]. The ASHI has been used in patients with osteoarthritis and rheumatoid arthritis.

The Disease Activity Score (DAS) and its modified version including 28 joint count (DAS28) have been developed to measure disease activity in patients with rheumatoid arthritis [75]. The DAS provides an absolute number that can be compared to other patients and to past and future scores in the same patient. Measures include a swollen joint count, tender joint count, acute-phase reactant (erythrocyte sedimentation rate or C-reactive protein), and patient assessment of global status. Pain assessment in the DAS is only indirect via the global status assessment.

## Conclusion

Animal models of different forms of arthritis have been developed for the assessment of knee joint pain. Limitations of their ability to mimic fully a condition as complex as arthritis in humans need to be considered carefully. Measurements of knee joint pain associated with arthritis in animal models include indirect (weight bearing, gait analysis, spontaneous mobility, and sensitivity of the paw to von Frey filaments or heat) and more direct measurements (probing the sensitivity of the knee, knee extension angle, and vocalizations evoked by stimulation of the knee). In patients with knee joint osteoarthritis or rheumatoid arthritis, physical measures include testing the mechanosensitivity of the knee, range of motion, weight bearing and gait analysis, whereas frequently used patient self-report questionnaires for pain assessment include the VAS and other rating scales, MPQ, WOMAC, BPI, HAQ, SF-36, and DAS.

## Abbreviations

ADL, activities of daily living

ASHI, Arthritis-Specific Health Index for the SF-36

CIA, collagen-induced arthritis

CFA, complete Freund's adjuvant

CLINHAQ, clinical HAQ

DAS, Disease Activity Score

HAQ, Health Assessment Questionnaire

HAQ-DI, Health Assessment Questionnaire Disability Index

K/C, kaolin-carrageenan

MDHAQ, multidimensional HAQ

MIA, monosodium iodoacetate

MPQ, McGill Pain Questionaire

NPS, Neuropathic Pain Scale

PPI, Present Pain Intensity

PWL, paw withdrawal latency

PWT, paw withdrawal threshold

SF-36, Medical Outcomes Study 36-Item Short-Form Health Survey

SF-MPQ, Short-Form McGill Pain Questionnaire

VAD, vocalization afterdischarge

VAS, Visual Analog Scale

VDS, vocalization during stimulation

WOMAC, Western Ontario and McMaster Universities Osteoarthritis Index

## Competing interests

VN and JSH hold a patent relating to the vocalization measurements in animals (U.S. Patent Application 98006/28). However, no royalties are being received and no financial gains are expected.

## Authors' contributions

VN reviewed the literature, gathered background information, and wrote the article. JSH, HA, GJ and YF assisted with literature review and conducted the behavioral experiments in Dr. Neugebauer's laboratory as described in this article.
